# Apoptotic CD8 T-lymphocytes disable macrophage-mediated immunity to *Trypanosoma cruzi* infection

**DOI:** 10.1038/cddis.2016.135

**Published:** 2016-05-19

**Authors:** M P Cabral-Piccin, L V C Guillermo, N S Vellozo, A A Filardy, S T Pereira-Marques, T S Rigoni, W F Pereira-Manfro, G A DosReis, M F Lopes

**Affiliations:** 1 Instituto de Biofísica Carlos Chagas Filho, Centro de Ciências da Saúde, Universidade Federal do Rio de Janeiro, Avenida Carlos Chagas Filho 373, CCS-IBCCF, Ilha do Fundão, Rio de Janeiro, RJ, Brazil; 2 Instituto Nacional para Pesquisa Translacional em Saúde e Ambiente na Região Amazônica, Conselho Nacional de Desenvolvimento Científico e Tecnológico/MCT, Rio de Janeiro, RJ, Brazil

## Abstract

Chagas disease is caused by infection with the protozoan *Trypanosoma cruzi*. CD8 T-lymphocytes help to control infection, but apoptosis of CD8 T cells disrupts immunity and efferocytosis can enhance parasite infection within macrophages. Here, we investigate how apoptosis of activated CD8 T cells affects M1 and M2 macrophage phenotypes. First, we found that CD8 T-lymphocytes and inflammatory monocytes/macrophages infiltrate peritoneum during acute *T. cruzi* infection. We show that treatment with anti-Fas ligand (FasL) prevents lymphocyte apoptosis, upregulates type-1 responses to parasite antigens, and reduces infection in macrophages cocultured with activated CD8 T cells. Anti-FasL skews mixed M1/M2 macrophage profiles into polarized M1 phenotype, both *in vitro* and following injection in infected mice. Moreover, inhibition of T-cell apoptosis induces a broad reprogramming of cytokine responses and improves macrophage-mediated immunity to *T. cruzi*. The results indicate that disposal of apoptotic CD8 T cells increases M2-macrophage differentiation and contributes to parasite persistence.

Chagas disease, caused by the pathogenic protozoan *Trypanosoma cruzi*, is endemic in Latin America, despite efforts to control insect vector and transmission.^1^ *T. cruzi* parasites infect virtually any cell, reaching the cytoplasm, where replication ensues, followed by cell death and spread of infective parasites. Most patients remain asymptomatic, but persistence of parasites often results in chronic disease, affecting the peripheral nervous system or the heart of patients.^2, 3^ Cellular and humoral immune responses overcome acute infection, but fail to eliminate *T. cruzi* and infected cells.^4^ No vaccine or effective drug for treating established disease is available.^3^

The protozoan *T. cruzi* is equipped with a molecular apparatus that triggers multiple Toll-like receptors and induces innate immunity. In particular, macrophages have a critical role as host cells, antigen-presenting cells, and effectors for parasite killing.^5, 6, 7, 8^ Depending on stimuli, macrophages can express distinct activation phenotypes and effects on infection. M1/classically activated/M (LPS+IFN-*γ*) macrophages usually produce IL-12 and NO for killing of intracellular pathogens, whereas alternatively activated macrophages, belonging to the M2 spectrum, help tissue repair and actually promote infection and tumor growth.^9, 10, 11, 12^

T cells as well as antibodies are required to control infection.^13, 14, 15^ CD8 T cells eliminate cells harboring parasites in the cytoplasm, whereas both CD4 and CD8 T cells produce IFN-*γ* and activate macrophages to restrain infection.^16, 17^ Effector T-lymphocytes also promote immunopathology in the heart.^18, 19^ Conversely, regulatory mechanisms, provided by cytokines and apoptosis of effector cells, dampen inflammation and prevent pathology, but might contribute for parasite persistence.^20, 21^

Lymphocytes undergo apoptosis in the course of *T. cruzi* infection, negatively affecting T-cell expansion,^22, 23^ B-cell response,^24^ parasite killing by classically activated macrophages,^23, 25^ and CD8 T-cell-mediated immunity.^26, 27^ Furthermore, uptake of apoptotic cells promotes infection within macrophages, through production of prostaglandin E-2 (PGE-2), TGF-*β*, and polyamines,^28^ features associated with M2/alternatively activated macrophages.^9^ By contrast, blockade of PGE-2 production or inhibition of lymphocyte apoptosis by caspase inhibitors reduces parasite growth *in vitro* and parasitemia.^28, 29^ Therefore, apoptosis and efferocytosis stand as potential therapeutic targets.^20, 30, 31^ However, it remains unknown whether efferocytosis affects M1/M2 macrophage polarization under the inflammatory setting of infection.

We investigated the molecular mechanisms underlying T-cell apoptosis in *T. cruzi* infection and found functional expression of both Fas (CD95) and Fas ligand (FasL),^23, 26^ caspase-8 activity, and activation of effector caspase-3^29^^,^^32^ in T cells from *T. cruzi*-infected mice. To prevent apoptosis during acute infection, we used both pharmacological approaches and apoptosis-defective mouse models.^23, 26, 29, 32^ However, intrinsic defects of immune responses have precluded accurate interpretations of immune outcomes in FasL-mutant mice^23^ or in mice expressing the caspase-8 inhibitor viral-FLIP (viral FLICE/caspase-8 inhibitory protein) in T cells.^32^ So far, the general caspase inhibitor zVAD and the antagonist anti-FasL mAb effectively inhibited apoptosis and potentiated immunity to *T. cruzi* infection.^26, 29^ CD8, but not CD4, T cells seem to be preferential targets for early effects of apoptosis inhibition in acute infection, based on the earlier kinetics of CD8 T-cell activation and higher Fas expression.^26, 29^ Both treatments improved CD8 T-cell survival, macrophage activation, and parasite control in infected mice.^26, 29^ The interactions between CD8 T cells and macrophages have not been fully explored and might be crucial to explain effective immunity induced by inhibition of apoptosis. Moreover, it is worth investigating how activated and apoptotic CD8 T cells affect distinct phenotypes of macrophages during infection. By using primary cultures of CD8 T cells and macrophages from infected mice, we recapitulated most features observed *in vivo* upon inhibition of apoptosis. As assessed by *in vitro* and *in vivo* approaches, inhibition of T-cell apoptosis affects the outcome of CD8 T-cell-macrophage crosstalk to reprogram the immune response to *T. cruzi* infection.

## Results

### CD8 T cells and monocytes repopulate peritoneal cavity in *T. cruzi* infection

**Figure 1:**
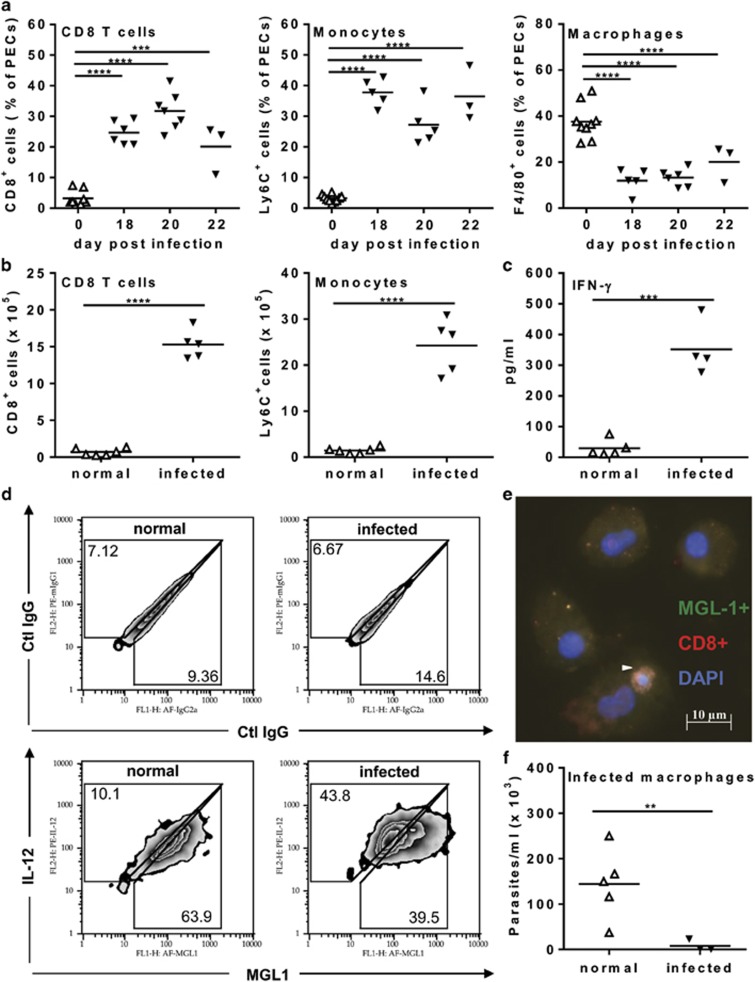
CD8 T cells and monocyte-derived macrophages in acute *T. cruzi* infection. (**a**) Percentages of CD8^+^ T cells, Ly6C^+^ monocytes, and F4/80^+^ macrophages in PECs from *T. cruzi-*infected mice compared with PECs from uninfected (d 0) mice. (**b**) Absolute numbers of CD8^+^ T cells and Ly6C^+^ monocytes and (**c**) IFN-*γ* in peritoneal exudates from normal and infected (18 dpi) mice. (**d**) Expression of MGL1 (M2) and IL-12p35 (M1) markers (as well as the respective control mAbs, upper panels) in F4/80^+^ macrophages from normal or infected (18 dpi) mice cultured during 48 h. (**e**) Image depicts PECs from infected (18 dpi) mice and represents results of three independent experiments. T cells were stained with anti-CD8 (PE, red, arrow head), macrophages stained with anti-MGL1 (Alexa Fluor 488, green), and nuclei marked with DAPI (blue). (**f**) Parasite burden as trypomastigotes released by macrophages from normal or infected (18 dpi) mice cultured during 3 weeks after infection with metacyclic parasites. In (**a**, **b**, **c**, and **f**), each symbol represents an individual normal (Δ) or infected (▾) mouse. Significant differences between normal (*N*=5–10) and infected (*N*=3–7) mice are indicated (*), as analyzed by analysis of variance (ANOVA) with Dunnett's post-test (**a**) and *t*-test (**b**, **c**, and **f**). Graphs depicted in (**d**) are representative of at least three independent experiments with pools of cells from infected (18 dpi) mice, cultured in two to four technical replicates. IL, interleukin

**Figure 2:**
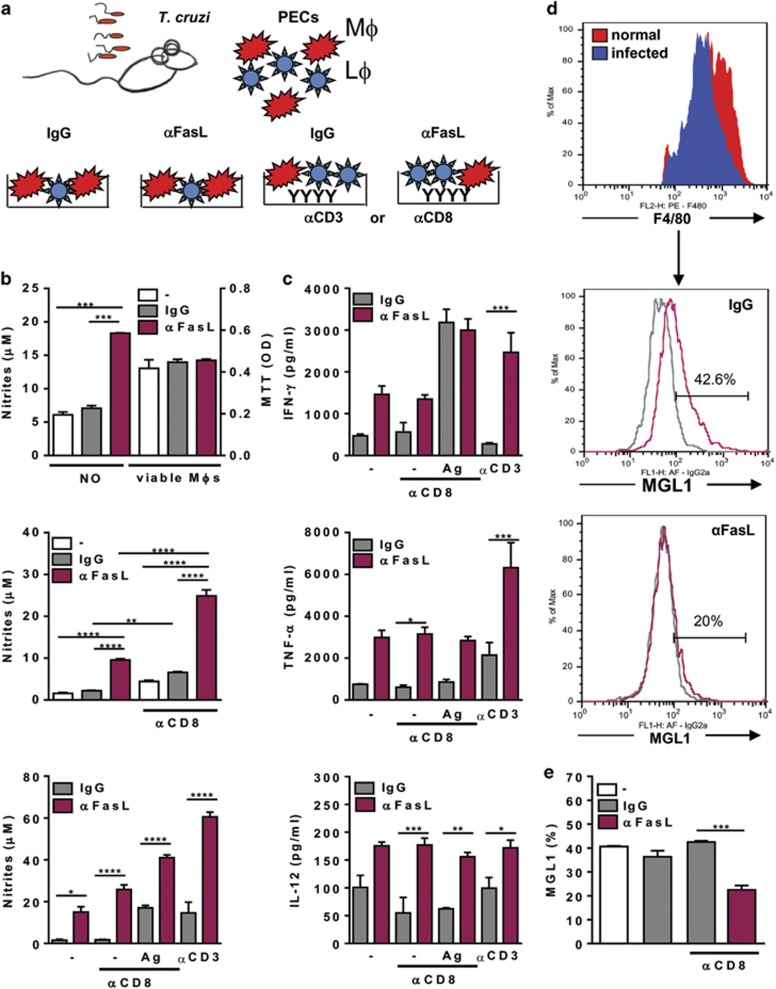
M2- to M1-phenotype shift in peritoneal cells treated with anti-FasL. (**a**–**f**) PECs from normal or *T. cruzi*-infected mice (18–20 dpi) were cultured with or without plate-bound anti-CD8 or anti-CD3 in the presence or absence of anti-FasL or control immunoglobulin G (IgG). (**b** and **c**) Some cultures were stimulated with *T. cruzi* Ag, as indicated. (**b**) NO production in supernatants and viability of adherent cells (by MTT assay) were evaluated after 24 h (**b**, upper panel) or 48 h. (**c**) secreted cytokines were measured in 48 h supernatants by enzyme-linked immunosorbent assay (ELISA). (**d** and **e**) Cells were detached for evaluation of MGL1 expression in gated F4/80^+^ cells. Results are expressed as means and S.E.M. and represent three independent experiments with three technical replicates of pooled cells from infected mice. Significant differences between treatments are indicated (*), as analyzed by *t*-test (**e**) or by analysis of variance (ANOVA) with Tukey's post-test (**b**, upper panel) or by two-way ANOVA with Tukey's (**b**, middle panel) or Bonferroni (**b,** lower panel, and **c**) post-test

We used BALB/c mice infected with chemically derived metacyclic trypomastigotes of the Dm28c *T. cruzi* clone, as a validated experimental model of Chagas disease.^33^ Parasitemia peaks ~3 weeks after infection and immune responses and inflammation in the heart reproduce features of *T. cruzi* infection induced by insect-derived metacyclic parasites.^33^ In addition, CD8 T cells predominate in heart inflammation,^33^ as observed in human patients.^34^ We assessed the phenotype of peritoneal exudate cells (PECs) during acute infection and found that about 20–40% of PECs are CD8 T cells (Figure 1a), whereas CD4^+^ cells represent only 5% of PECs in both normal and infected mice (not shown). Absolute numbers of CD8 T cells also increase during infection (Figure 1b). CD8 T cells represent an important source of IFN-*γ*, as detected in peritoneal exudates from infected mice (Figure 1c) and in PECs stimulated with a *T. cruzi* antigen (Ag; Tzelepis *et al.*^35^) (Supplementary Figure S1). Although 30–50% of PECs from normal mice express the F4/80 macrophage marker at high levels, only 10–20% of PECs from infected mice express F4/80 at intermediate levels (F4/80^int^) (Figure 1a). By contrast, 30–40% of PECs from infected, but not normal, mice bear the Ly6C marker of inflammatory monocytes/macrophages (Figure 1a). Absolute numbers of monocytes (Figure 1b), but not F4/80^+^ macrophages (not shown), also increase upon infection. F4/80^int^ macrophages from infected mice seem to be derived from Ly6C^+^ monocytes, instead of residual resident macrophages. Therefore, inflammatory monocytes/macrophages comprise about 40–60% of PECs, which adhere to culture plates and acquire F4/80^int^ (Figure 2d). About 90% of adherent cells are F4/80^int^ and CD11b-double positive macrophages.

### Macrophages from infected mice express M1 and M2 phenotypes

To address the functional phenotype of macrophages, we measured the expression of IL-12p35 and macrophage galactose-type lectin-1 (MGL1, CD301a)^36^ as surrogate M1 and M2 markers. Although most macrophages from normal mice express MGL1, IL-12p35 and MGL1 defined two subsets in macrophages from infected mice (Figure 1d). In addition, macrophages from normal mice are more permissive to parasite infection than macrophages from infected mice (Figure 1f). Interestingly, we detected MGL1^+^ macrophages associated with cell debris from apoptotic CD8 T cells in PECs from infected mice (Figure 1e and Supplementary Figure S2). Accordingly, 20–30% of peritoneal CD8 T cells undergo apoptosis during acute infection (not shown) and up to 40% of F4/80 macrophages bear intracellular CD8^+^ events (Supplementary Figure S2). These results suggest that the development of functional phenotypes by macrophages is under the influence of activation by type-1 cytokines *versus* suppression by apoptotic cells.

### Blockade of FasL affects the crosstalk between T cells and macrophages

We took advantage of the presence of both CD8 T cells and monocytes/macrophages in the peritoneum of infected mice to investigate the interactions between CD8 T cells and macrophages. We hypothesized that the presence of apoptotic cells could counterbalance macrophage activation and affect immunity to *T. cruzi* infection. We previously reported that efferocytosis enhances parasite growth within macrophages from *T. cruzi*-infected mice.^28^ To evaluate how apoptosis of activated T cells affects macrophage phenotype, we treated cultures with the neutralizing anti-FasL or control IgG mAb (Figure 2a). Anti-FasL increased NO responses, but did not improve macrophage viability (Figure 2b and Supplementary Figure S3). We also used plate-bound anti-CD8 to obtain CD8 T-cell enriched PECs from infected mice (70–80% from culture supernatants). Strikingly, treatment with anti-FasL induced NO in CD8 T-cell enriched cultures, both in the presence and absence of Ag stimulation (Figure 2b, lower panels). Anti-FasL potentiated IL-12 and TNF-*α*, but not IFN-*γ* response in CD8 T-cell enriched cultures (Figure 2c). Treatment with anti-FasL also increased production of NO (Figure 2b) and of type-1 cytokines (Figure 2c) in anti-CD3-activated cultures. Finally, anti-FasL reduced MGL1 expression in F4/80^+^ macrophages (Figures 2d and e). Therefore, treatment of primary cultures of peritoneal lymphocytes and macrophages with anti-FasL promoted M1-macrophage phenotype, but restrained M2-macrophages.

### Apoptosis in cocultured CD8 T cells disables macrophage-mediated immunity

**Figure 3:**
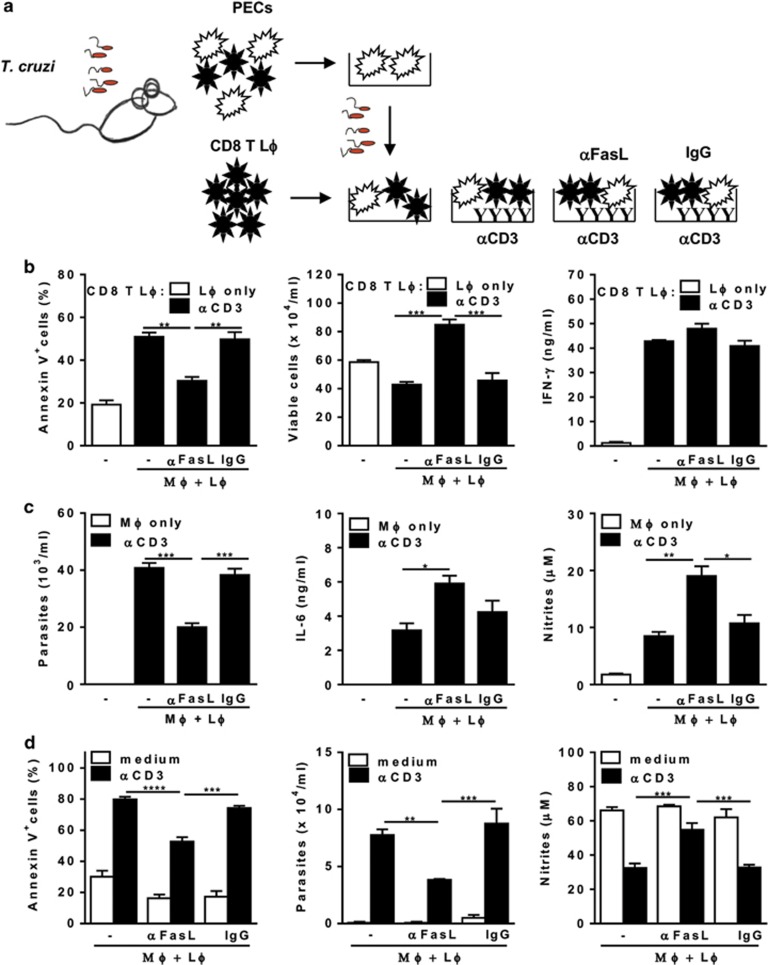
FasL-mediated apoptosis of CD8 T cells drives *T. cruzi* infection in cocultured macrophages. (**a–d**) Peritoneal macrophages from infected (18 dpi) mice were infected with *T. cruzi* and cocultured with purified splenic CD8 T cells from infected (18–20 dpi) mice in the presence of interleukin-2 (IL-2). Cocultures were stimulated or not with soluble anti-CD3 and treated or not with anti-FasL or control immunoglobulin G (IgG). (**b–d**) After 48 h, culture supernatants were collected for NO or cytokine responses. (**b** and **d**) CD8 T cells were collected for detection of apoptosis by annexin V and 7-aminoactinomycin D (7-AAD) staining. (**c** and **d**) Adherent cells were cultured during 2–3 weeks for determination of parasite burden, as released trypomastigotes. Data are expressed as means and S.E.M. of three technical replicates. Each set of data (**b–d**) represents at least three independent experiments. Significant differences between treatments in anti-CD3 activated cultures are indicated (*), as analyzed by analysis of variance (ANOVA) (**b** and **c**) or by two-way ANOVA (**d**) with Tukey's post-test

We investigated whether or not T-cell apoptosis affects macrophage activation, by coculturing macrophages with purified/splenic CD8 T cells from infected mice (Figure 3a). For infected cocultures, PECs from infected mice were carefully washed to remove non-adherent cells and then infected with metacyclic trypomastigotes. We used soluble anti-CD3 to induce activation and apoptosis of CD8 T cells. Anti-FasL, but not control IgG, reduced CD8 T-cell apoptosis (based on annexin V and 7-AAD staining), increasing by two times the recovery of viable CD8 T cells from cocultures, but not IFN-*γ* production by CD8 T cells (Figure 3b). Remarkably, parasites replicated in the presence of activated CD8 T cells, as opposed to cultures with macrophages only (Figure 3c), in spite of NO production in activated cocultures (Figure 3c). By contrast, treatment with anti-FasL helped macrophages to control infection and further increased macrophage activation, as assessed by IL-6 and NO production (Figure 3c). Anti-FasL did not increase IFN-*γ* in cocultures and neutralization of IFN-*γ* did not revert the effects of anti-FasL on NO production (not shown). In addition, we did not find any direct effect of anti-FasL on macrophages alone (not shown). To address any role of anti-FasL independent of preventing activation-induced apoptosis, unstimulated cocultures were also treated with anti-FasL or control IgG (Figure 3d). Apoptosis in CD8 T cells and parasite growth in macrophages were observed in anti-CD3-activated cocultures, whereas concomitant treatment with anti-FasL reduced apoptosis and *T. cruzi* infection (Figure 3d, left panels). Treatment with anti-FasL also improved NO production in stimulated cocultures (Figure 3d). Compared with control IgG, we did not find any significant effect of anti-FasL in unstimulated cocultures (Figure 3d). These results are compatible with previously observed deleterious effects of efferocytosis on NO production and parasite killing induced by direct activation with IFN-*γ* and LPS.^28^

### FasL-induced T-cell apoptosis reprograms macrophage responses

**Figure 4:**
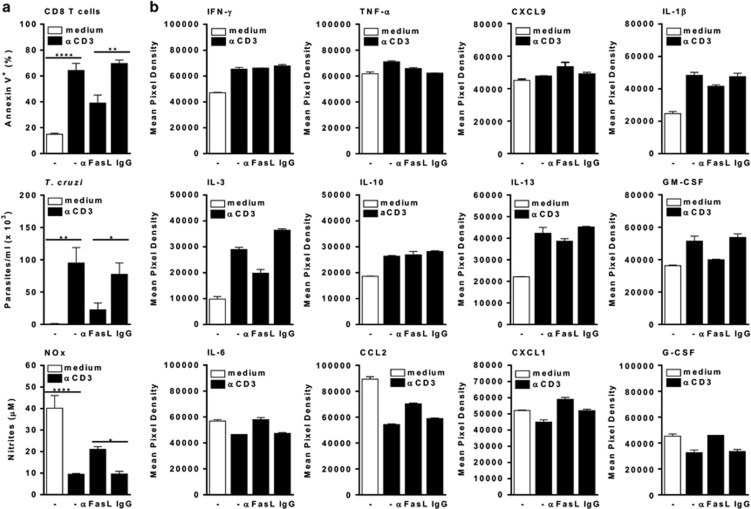
Apoptosis of CD8 T cells correlates with a broad reprogramming of macrophage cytokine responses. (**a** and **b**) Infected macrophages and splenic CD8 T cells from infected mice were stimulated or not with soluble anti-CD3 in the presence of interleukin-2 (IL-2). Anti-CD3-activated cocultures were treated or not with anti-FasL or control immunoglobulin G (IgG). After 48 h, culture supernatants were collected for evaluation of NO (**a**) or cytokine (**b**) responses, as assessed by a cytokine array. CD8 T cells were analyzed for annexin V/7-AAD (7-aminoactinomycin D) staining (**a**). Adherent cells were cultured during 4 weeks for determination of released trypomastigotes (**a**). Data represent mean and S.E.M. of two (**b**), or three to four (**a**) technical replicates. Data depicted in (**a**) represent at least three independent experiments. Significant differences between treatments are indicated (*), as analyzed by analysis of variance (ANOVA) with Bonferroni post-test (**a**)

**Figure 5:**
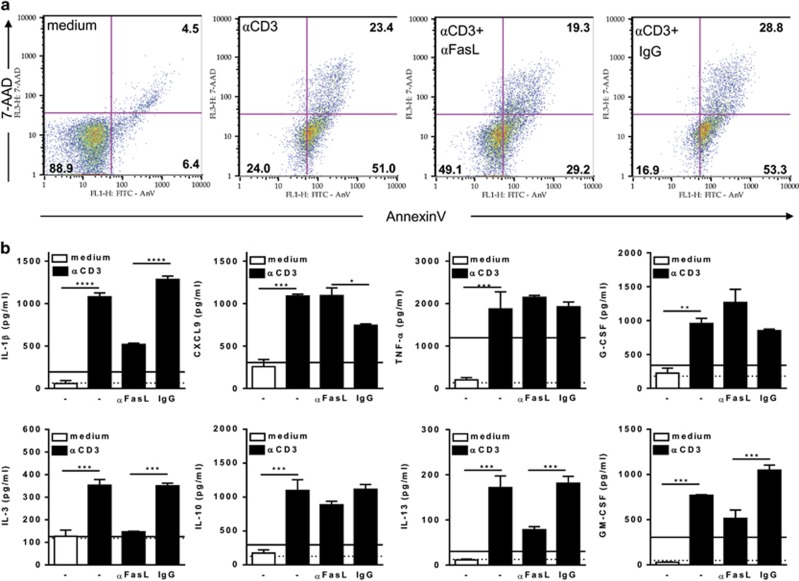
Apoptosis of CD8 T cells correlates with M2-cytokine responses. (**a** and **b**) Peritoneal macrophages and splenic CD8 T cells from infected mice were stimulated or not with soluble anti-CD3 in the presence of interleukin-2 (IL-2). Anti-CD3-activated cocultures were treated or not with anti-FasL or control immunoglobulin G (IgG). After 48 h, culture supernatants were collected for evaluation of cytokine responses, as assessed by enzyme-linked immunosorbent assay (ELISA) (**b**). CD8 T cells were analyzed for annexin V/7-AAD (7-aminoactinomycin D) staining (**a**). (**b**) Full lines stand for cytokines produced by stimulated CD8 T cells and dotted lines represent cytokine responses by macrophages. Data represents mean and S.E.M. of three to four technical replicates in at least three independent experiments. Significant differences between treatments are indicated (*), as analyzed by analysis of variance (ANOVA) with Bonferroni post-test

The functional phenotype of infected macrophages cocultured with CD8 T cells was also assessed by cytokine array and ELISA assays. Stimulation of CD8 T cells by anti-CD3 triggered apoptosis, promoted parasite growth, reduced NO production (Figure 4a), and induced a reprogramming of macrophage phenotype (Figure 4b). In the absence of anti-CD3, cocultured (infected) macrophages expressed a predominant M1 phenotype, by producing NO (Figure 4a) as well as type-1 cytokines (Figure 4b, upper and lower panels). We were able to identify at least two distinct patterns of responses to activation with anti-CD3 and treatment with anti-FasL. First, anti-CD3 downmodulated NO and M1 cytokines (Figures 4a and b, lower panels). Second, a new set of cytokines (IL-1*β*, IL-3, IL-10, IL-13, and GM-CSF) was upregulated (Figure 4b). Remarkably, treatment with anti-FasL prevented activation-induced apoptosis, reduced parasite growth, and partially restored NO production as well as the M1 phenotype (Figures 4a and b, lower panels). By contrast, IL-3, IL-13, and GM-CSF were downmodulated upon anti-FasL treatment (Figure 4b). Only low levels of other type-1 (IL-12p70, IL-23) or type-2 (CCL11, CCL17, IL-4, IL-5) responses were detected (not shown). To rule out the effects of parasites on cytokine responses, we further assessed treatment with anti-FasL to prevent apoptosis in cocultures that were not exposed to *T. cruzi in vitro* (Figure 5a). Different from infected cocultures, type-1 cytokines were at basal levels in unstimulated cocultures (Figure 5b). Nonetheless, secretion of CXCL9, TNF-*α*, and G-CSF increased upon anti-CD3 stimulation (Figure 5b). Similarly, IL-1*β*, IL-3, IL-10, IL-13, and GM-CSF were upregulated in activated cocultures. Treatment with anti-FasL reduced the levels of IL-1*β*, IL-3, IL-13, and GM-CSF, but increased CXCL9 secretion (Figure 5b). Therefore, activation-induced T-cell apoptosis induces macrophages to express an M2-like phenotype that is unable to control *T. cruzi* infection.

### Anti-FasL reduces M2 macrophages in *T. cruzi* infection

**Figure 6:**
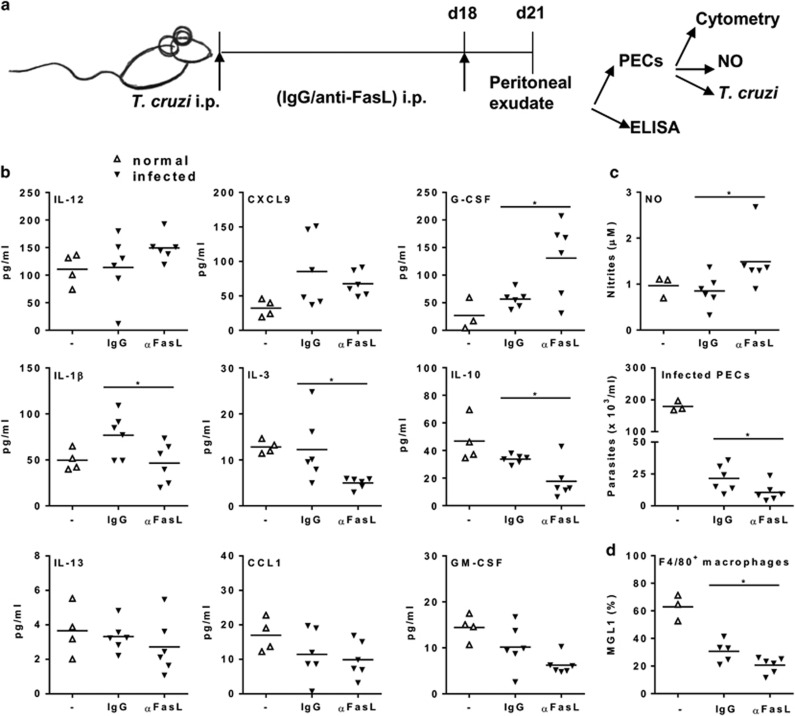
Treatment with anti-FasL promotes macrophage-mediated immunity to *T. cruzi* infection. (a–d) Infected BALB/c mice (18 dpi) were treated intraperitoneally with anti-FasL (100 *μ*g) or control immunoglobulin G (IgG). Uninfected mice were used as controls. (**b**) After 3 days, peritoneal exudates were analyzed for cytokines and chemokines. (**c**) Peritoneal macrophages were cultured during 24 h before evaluation of spontaneous NO production or were infected with *T. cruzi* and cultured during 2 weeks before determination of parasite burden. (**d**) PECs were evaluated for MGL1 expression in F4/80^+^ cells. Symbols represent peritoneal cells or exudates from individual normal (Δ) or infected (▾) mice treated with anti-FasL or immunoglobulin G (IgG) (*N*=6 mice per group). In (**c**), each symbol represents the means of two to three technical replicates of cultured cells from each individual mouse. Significant differences between treatments are indicated as (*) for *P*<0.05 in *t*-test

We investigated whether treatment *in vivo* with anti-FasL would be effective enough to modulate macrophage phenotype in the activated setting of infection. We evaluated macrophage phenotype 3 days after a single intraperitoneal injection of anti-FasL in infected mice (Figure 6a). Compared with mice that received control IgG, mice treated with anti-FasL showed increased production of G-CSF, but reduced secretion of IL-1*β*, IL-3, IL-10 (Figure 6b), and expression of the M2 marker MGL1 in F4/80^+^ macrophages (Figure 6d). Moreover, spontaneous NO production increased in 24 h cultured macrophages, whereas parasite growth decreased in macrophages from mice treated with anti-FasL (Figure 6c). Therefore, treatment with anti-FasL replaced mixed M1/M2 cells with effector M1 macrophages in infected mice. These results indicate that the blockade of lymphocyte apoptosis by anti-FasL improves macrophage-mediated immunity, but do not rule out the inhibition of apoptosis in other cells or other possible effects of blocking FasL during infection.

### Caspase inhibition increases M1/M2 macrophage ratio

**Figure 7:**
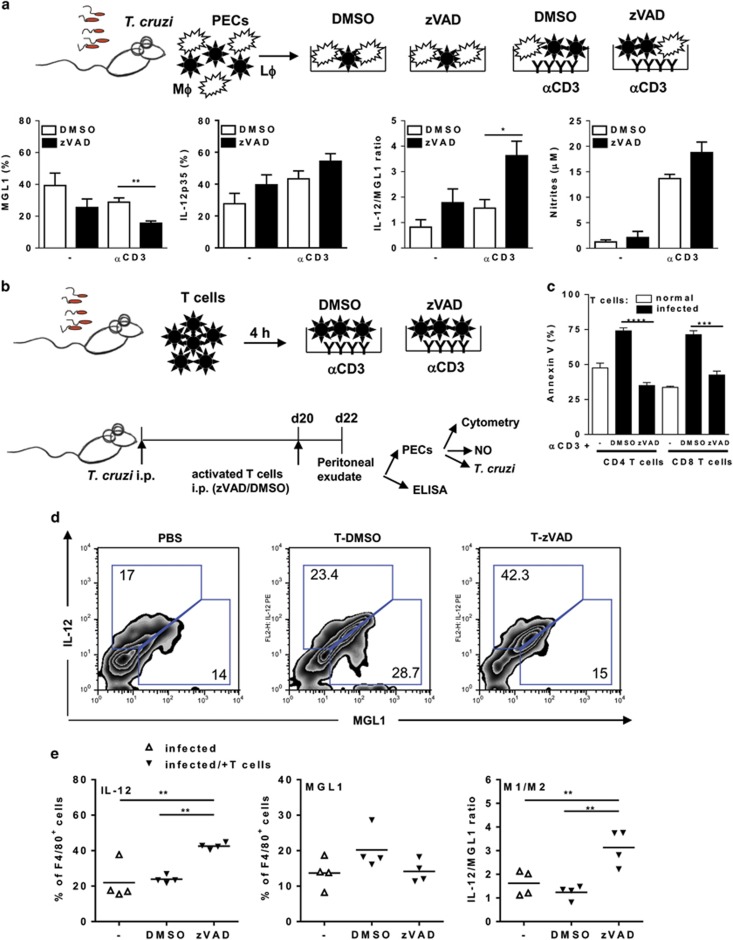
Inhibition of activation-induced T-cell death shifts M2 into M1 macrophages. (**a**) PECs from *T. cruzi-*infected mice were cultured with or without plate-bound anti-CD3 in the presence of caspase inhibitor zVAD or dimethyl sulfoxide (DMSO). Cells were detached after 48 h for evaluation of MGL1 and IL-12p35 expression in gated F4/80^+^ cells. NO production was measured in 48 h culture supernatants. Results are expressed as means and S.E.M. of three to four technical replicates of pooled PECs from infected mice. (**b**) T cells from normal or infected mice (20 dpi) were treated with anti-CD3 in the absence or presence of zVAD or DMSO during 4 h for *in vivo* injection and 24 h (**c**) for flow cytometry. (**c**) T cells were analyzed for annexin V staining in CD8 and CD4 T cells. Results are expressed as means and S.E.M. of four to five technical replicates. (**d** and **e**) Infected mice (20 dpi) were injected intraperitoneally with phosphate-buffered saline (PBS) (−) or with 2 × 10^6^ activated T cells (treated with zVAD or DMSO). After 2 days, PECs were collected and activated with PMA and ionomycin, before staining for MGL1 and IL-12p35 expression in F4/80^+^ cells. (**e**) Symbols represent PECs from individual infected mice treated with PBS (Δ) or with T cells (▾) activated in the presence of zVAD or DMSO (*N*=4 mice per group). Significant differences between treatments are indicated (*), as analyzed by *t*-test (**a** and **c**) or by analysis of variance (ANOVA) with Tukey's post-test (**e**)

**Figure 8:**
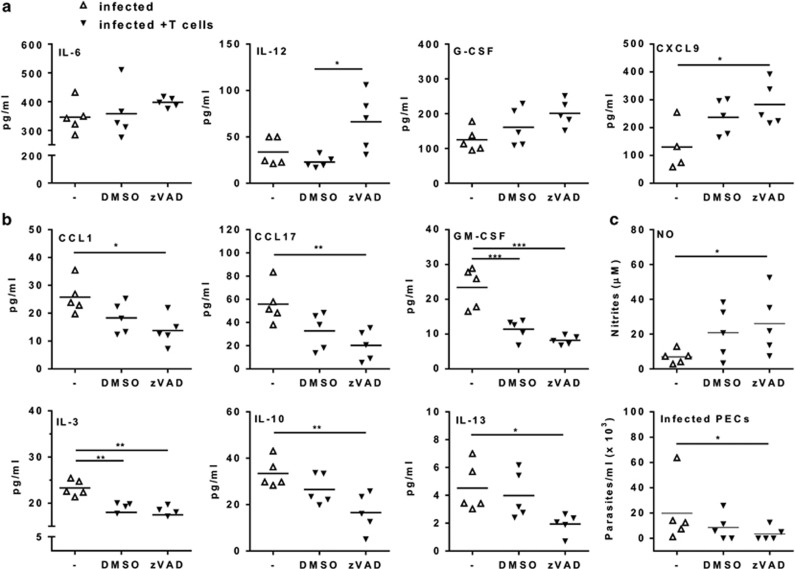
Inhibition of T-cell apoptosis enhances macrophage-mediated immunity to *T. cruzi* infection. (**a**–**c**) Splenic T cells from infected mice (18 dpi) were treated with anti-CD3 in the presence of caspase inhibitor zVAD or dimethyl sulfoxide (DMSO) for 4 h. T cells were washed and adoptively transferred intraperitoneally to infected mice (20 dpi). Infected mice injected with phosphate-buffered saline (PBS) only were used as controls. After 2 days, peritoneal exudates were analyzed for (**a**) M1 and for (**b**) M2 cytokines. (**c**) Peritoneal macrophages were cultured during 24 h before evaluation of spontaneous NO production or infected with *T. cruzi* and cultured during 4 weeks before determination of parasite burden. (**a** and **b**) Symbols represent peritoneal exudates from individual mice injected with PBS (Δ) or with activated T cells (▾) previously treated with zVAD or DMSO (*N*=5 mice per group). In (**c**), each symbol represents the mean of two to three technical replicates of cultured cells from each individual mouse. Significant differences between treatments are indicated (*), as analyzed by *t*-test (**c**) or by analysis of variance (ANOVA) with Tukey's post-test (**a** and **b)**

We used the general caspase inhibitor zVAD.fmk to target intracellular caspases.^29^ Treatment with zVAD reduces caspase-3 activation and apoptosis in CD8 T cells from infected mice (Silva *et al.*^29^ and data not shown). Moreover, pretreatment with zVAD prevented efferocytosis of CD8 T cells by cocultured macrophages (Supplementary Figure S2). To evaluate how blockade of T-cell apoptosis affects macrophage phenotype, we used *in vitro* and *in vivo* approaches (Figures 7a and b). First, plate-bound anti-CD3 captured (and activated) peritoneal T cells together with adherent PECs. Then, we added zVAD or DMSO to these primary cultures of T cells and macrophages or to macrophage cultures (Figure 7a). Compared with control diluent (DMSO), treatment with zVAD reduced MGL1 expression and doubled the IL-12/MGL1 ratio in F4/80^+^ macrophages from activated cultures (Figure 7a). Therefore, treatment of activated cultures of peritoneal lymphocytes and macrophages with zVAD altered macrophage phenotype. We did not find significant effects of zVAD on macrophages alone. For *in vivo* experiments, splenic T cells (both CD4 and CD8 T cells at a 1:1 ratio) from infected mice were first stimulated with anti-CD3 in the presence of zVAD or DMSO for 4 or 24 h (for apoptosis assays, Figure 7c). After 4 h, activated T cells were washed and injected in infected mice (Figures 7d and e). Controls were infected mice injected with PBS. After 2 days, PECs were collected and activated, before staining for MGL1 and IL-12p35 expression in F4/80^+^ cells. Following injection of activated T cells treated with zVAD, F4/80^+^ macrophages from infected mice expressed two times more IL-12p35 and increased IL-12p35/MGL1 ratio (Figures 7d and e), as well as IL-12p70 (Figure 8a), compared with mice injected with DMSO-treated T cells. Peritoneal exudates from recipients of zVAD-treated T cells also expressed increased levels of CXCL9 (Figure 8a) and reduced secretion of M2 cytokines (Figure 8b) in comparison with infected mice injected with PBS. Furthermore, cultured macrophages from mice that received zVAD-treated T cells produced more NO and showed better control of *in vitro* infection than macrophages from mice injected with PBS (Figure 8c). Therefore, T-cell apoptosis has a broad effect on macrophage functional phenotype and we were able to restore macrophage-mediated immunity by using strategies that prevent T-cell apoptosis both *in vitro* and *in vivo*.

## Discussion

Here, we addressed how the interactions between macrophages and CD8 T cells affect macrophage phenotype and ability to fight parasite infection. We found that FasL-mediated apoptosis of CD8 T cells induces a broad reprogramming of macrophage responses and is a promising target for upregulation of immune responses to infection. We show that interventions with agents that block T-cell apoptosis and efferocytosis (i.e., anti-FasL or zVAD) restore macrophage-mediated immunity.

Previously, we used earlier and repeated injections of anti-FasL or zVAD, which improved both lymphocyte and macrophage immune responses, and reduced parasitemia.^26, 29^ Here we tested a delayed and shorter treatment during acute infection, by the timing that CD8, but not CD4, T cells reach inflammatory macrophages in peritoneal cavity. Inflammatory macrophages from infected mice express Ly6C, a monocyte marker also found in immature myeloid cells from the hearts of *T. cruzi*-infected mice.^37^ Therefore, during acute infection, peritoneal macrophages bear features of immature myeloid cells, and only a fraction of PECs express F4/80. Upon adherence, PECs from infected mice lose Ly6C and acquire F4/80^int^ expression, along with M1 (IL-12) and M2 (MGL1) markers. Nonetheless, primary cultures of macrophages and peritoneal CD8 T cells treated with anti-FasL or zVAD had reduced M2 and increased M1 phenotype. Moreover, co-treatment with anti-FasL exacerbated type-1 responses to both anti-CD3 and parasite Ag.

In cultures of macrophages and splenic CD8 T cells from infected mice, macrophages express NO-producing M1 phenotype and parasite killing. By contrast, activation with anti-CD3 induced apoptosis of CD8 T cells, reduced NO production, and markedly increased the number of parasites, in spite of higher levels of IFN-*γ* in cocultures. Importantly, co-treatment with anti-FasL improved recovery of viable CD8 T cells and NO production and helped macrophages to control parasite infection. Therefore, increased parasite replication correlated with the onset of apoptosis in CD8 T cells.

Next, we focused on the cytokines modulated by both anti-CD3 and anti-FasL. We found that activation and treatment with anti-FasL led to a broad reprogramming of macrophage response. Anti-FasL upregulated the proinflammatory cytokines IL-6 and CXCL9, but reduced IL-1*β*, IL-3, IL-13, and GM-CSF. Although the production of IL-1*β* might help immunity to *T. cruzi*,^38^ IL-1*β* secretion did not correlate with NO production or with parasite killing in cocultures. By contrast, parasite growth correlated with production of IL-3, IL-13 (along with IL-10), and GM-CSF, which are potential parasite promoters, either by inducing PGE-2^39^ and arginase^40^ or by directly affecting parasite growth,^25^ features associated with M2 macrophages. Similarly, M2 macrophages induced in the absence of IL-12 promote infection with *T. cruzi*.^10^ Therefore, by blocking apoptosis of CD8 T cells in cocultures, treatment with anti-FasL restricted an M2-like phenotype and parasite growth and increased NO and other M1 features. Similarly, short-term treatment with anti-FasL *in vivo* reduced M2 responses, but improved parasite killing by macrophages. Therefore, the blockade of lymphocyte apoptosis with anti-FasL or zVAD correlates with upregulated M1/M2 ratio and promotes macrophage-mediated immunity. Furthermore, based on the myriad of cytokines and chemokines^11^ involved, it could have a broad impact on immune responses.

To our knowledge, we are the first to show that apoptosis of activated CD8 T cells directly affects the macrophage phenotype and is a potential target for improvement of immunity. Although both zVAD and anti-FasL were effective to boost immunity, as opposed to caspase inhibition, the Fas-death pathway stands as a more selective target. This is important to prevent potential side effects of blocking apoptosis or efferocytosis, such as breaking self-tolerance and regulation of immune responses, disrupting tissue repair or affecting immunity to tumors.^41, 42, 43^ By contrast, treatment with anti-FasL might affect apoptosis during ongoing immune responses that lead to upregulation of FasL. Nonetheless, other direct or indirect effects of anti-FasL on macrophage activation^44^ or death^45^ could not be excluded, and potential increase of subsequent Th1 and Th2 responses^23, 26^ should be taken into account.

Recently, Vasconcelos *et al.*^27^ showed that the proapoptotic phenotype of Ag-specific CD8 T cells expressing higher levels of Fas correlates with defective immune responses to *T. cruzi* infection. Moreover, a virus vaccine expressing a parasite Ag prevented the development of proapoptotic CD8 T cells and promoted immunity, comprising both cytotoxic and cytokine-expressing CD8 T cells.^27^ Based on these and previous findings, we consider two major, but not exclusive, hypotheses to explain modulation of parasite infection by treatment with anti-FasL. First, by preventing CD8 T-cell death, anti-FasL might restrain the negative effects of efferocytosis on macrophage activation. Second, anti-FasL would help immunity by rescuing effector CD8 T cells, which either activate or kill macrophages from *T. cruzi*-infected mice. Although we have evidence of both cytotoxic activity and IFN-*γ* production by CD8 T cells, the effects of anti-FasL on macrophage phenotype do not correlate with higher IFN-*γ* levels or with increased macrophage viability or with reduced cytotoxicity. It is worth investigating whether blockade of apoptosis promotes heightened effector activities by antigen-specific CD8 T cells *in vivo* and how treatment with anti-FasL or zVAD affects distinct subsets of effector cells. Importantly, perforin- and IFN-*γ*-expressing subsets of CD8 T cells might have opposing (pathogenic *versus* protective) roles in Chagas disease.^18^

Our results are compatible with the hypothesis that inhibition of CD8 T-cell apoptosis potentiates macrophage-mediated immunity by restraining the inhibitory effects of efferocytosis on macrophages. First, apoptotic cells have direct effects on macrophages and on infected mice to promote parasite,^25, 28, 46^ bacterial,^47, 48, 49, 50^ and viral^51^ infections. Second, efferocytosis changes macrophage function by inducing distinct metabolic programs^28, 50^ and production of soluble factors.^28, 48, 49, 50^ Efferocytosis induces secretion of TGF-β,^28^ which suppresses induced-nitric oxide synthase expression (Supplementary Figure S4) and NO production^28^ upon LPS and/or IFN-*γ* stimulation. Third, the effects of apoptotic cells can be reverted by interfering with the uptake of apoptotic cells, the metabolic activity, or the production of soluble mediators.^28, 48, 49^ Nonetheless, it is noteworthy that either activation or suppression of immune responses ensues when efferocytosis is mediated by dendritic cells^52, 53^ or by macrophages phagocytosing apoptotic cells, depending on the genetic background/the immune features of cells^46^ or infections.^50, 53, 54^ Recent findings suggest that macrophages use different sets of receptors to efferocytose under homeostatic or inflammatory conditions.^55^ The experimental model of Chagas disease provides an opportunity to investigate how different receptors are involved in efferocytosis and modulation of macrophage phenotype under the inflammatory setting of *T. cruzi* infection as well as how this will affect the development of disease. Whereas M1 macrophages might promote inflammation, M2 macrophages can induce fibrosis in the heart^56^ in the context of parasite infection.

Here we show that whereas effector CD8 T cells might contribute to the inflammatory environment in tissues, apoptosis of CD8 T-lymphocytes counterbalances macrophage activation, negatively affecting immunity to *T. cruzi* infection, by promoting macrophage differentiation towards an M2-like phenotype. Moreover, pharmacologic tools that target apoptosis are able to restore M1-macrophage responses, opening new avenues to promote immunity to infectious diseases.

## Materials and Methods

### Mice and *T. cruzi* infection

BALB/c mice were obtained from the Fluminense Federal University (UFF) or from the Oswaldo Cruz Institute (FIOCRUZ) and maintained in the animal facility at the Federal University of Rio de Janeiro (UFRJ). Male BALB/c mice, aged 6–8 weeks, were infected intraperitoneally with 2 × 10^5^ metacyclic trypomastigotes of Dm28c *T. cruzi* clone, obtained by chemically induced metacyclogenesis in triatomine artificial urine-proline medium.^33^ Experiments were conducted during acute (18–22  days post infection (dpi)) infection and age-matched BALB/c mice were used as uninfected controls. All procedures were approved (protocol IBCCF178) by the University's Ethical Committee for Animal Experimentation (UFRJ), according to national and institutional regulations that comprise with international standards.

### Flow cytometry assays

PECs or cultured cells were washed in FACS buffer (plus 2% FCS), followed by incubation with anti-CD16/CD32 (eBioscience, San Diego, CA, USA) for Fc blocking. We stained cells with anti-CD8, anti-CD5, anti-CD19, anti-B220, anti-F4/80 mAbs, anti-CD11b, and anti-CD11c (clone HL3) labeled with PE, FITC, or allophycocyanin (BD Biosciences, Chicago, IL, USA), or with FITC-labeled anti-Ly6G (clone 1A8), allophycocyanin-labeled anti-Ly6C (clone HK1.4), or anti-CD4 mAbs (eBioscience), or with Alexa Fluor 488-labeled anti-MGL1 (CD301a) mAb (AbD Serotec, Kidlington, UK) or control rat IgG2a mAb (R&D Systems, Minneapolis, MN, USA). For intracellular staining, we washed, permeabilized, and stained cells with PE-labeled anti-IL-12p35 or control murine IgG1 mAb (R&D Systems). For IL-12p35 (M1) and MGL1 (M2) subsets, gates were based on the exclusion of background staining with isotype control mAbs. We then washed, fixed, and acquired cells with the CellQuest software on a FACSCalibur system (BD Biosciences). For apoptosis detection, cells were first stained with allophycocyanin anti-CD8, washed, and then treated with FITC-annexin V (BD Biosciences) for 20 min at room temperature, according to the manufacturer; 7-AAD (eBioscience) was added just before flow cytometry. For analysis, we used the FlowJow software (TreeStar, Ashland, OR, USA).

### Microscopy

Upon cytospining, PECs were fixed in a glass layer with 4% paraformaldehyde, followed by staining with Alexa Fluor 488-labeled anti-MGL1, PE-labeled anti-CD8, and DAPI (4′,6-diamidino-2-phenylindole, dihydrochloride; Invitrogen Life Technologies, Carlsbad, CA, USA) for nuclear staining. For immunofluorescence, images were captured with an Imager M2 ApoTome microscope (Zeiss, Oberkochen, Germany) or with an EVOS_fl_ (AMG – Advanced Microscopy Group, Bothell, WA, USA). For optical microscopy, PECs from infected mice were stained with the Panoptic Staining Kit (Laborclin, Pinhais, Brazil). The images were acquired with an Olympus BX51 microscope (Olympus Optical Co., Tokyo, Japan) and an Olympus 72 digital camera (Olympus Optical Co.).

### Peritoneal exudates and cell cultures

PECs were collected in 3–5 ml of DMEM (Invitrogen Life Technologies), supplemented with 2 mM glutamine, 5 × 10^5^ M 2-ME, 10 *μ*g/ml gentamicin, 1 mM sodium pyruvate, and 0.1 mM MEM nonessential amino acids (culture medium). Upon centrifugation, supernatants were collected for cytokine assays. PECs were processed for flow cytometry and cultures. PECs (1 or 1.5 × 10^6^ cells) were added to 24-well vessels and maintained in culture medium plus 10% FCS (Invitrogen Life Technologies) during 2–3 h and then gently washed to remove the excess of non-adherent cells, but retain CD8 T cells (30–40% of cells recovered from 24 h supernatants washed from cultures). Other non-adherent PECs are about 14–20% of B cells, 5–7% CD4 T cells, 1–2% CD11c^+^, and 1–4% Ly6G^+^ cells. Cultures enriched in T cells (70–80% CD8 T cells recovered from culture supernatants) were obtained by culturing PECs on plate-bound anti-CD8 (53–6.7) mAb or anti-CD3 (145-2C11) mAb (10 μg/ml; BD Biosciences) during 2 h before washing out non-adherent cells. The remaining cells were maintained in culture medium plus 10% FCS at 37 °C and 7% CO_2_ in a humid atmosphere. Some cultures also received *T. cruzi* Ag (lysates of 1 × 10^6^ metacyclic parasites per well). These mixed cultures were treated or not with 10 *μ*g/ml of anti-FasL (MFL3) mAb or control IgG mAb (eBioscience), or with 40 *μ*M of zVAD (BD Biosciences) or 0.2% DMSO (Sigma, St Louis, MO, USA). After 48 h, we collected supernatants for cytokine assays or mixed supernatants with equal volumes of the Griess reagent (1% sulfanilamide, 0.1% naphthylethylenediamine dihydrochloride, 2% H_3_PO_4_; Sigma) to determine NO production. The absorbance was measured at 540 nm and results are expressed as the content of nitrites, based on the concentration of sodium nitrite solutions as standard. After washing out non-adherent cells, macrophages were evaluated for viability by MTT (3-(4,5-dimethylthiazol-2-yl)-2,5-diphenyltetrazolium bromide from Sigma) assay or detached with 2 mM EDTA (plus 2% FCS) at 4 °C and processed for flow cytometry, as above.

### Infected macrophages

PECs (5 × 10^5^ or 1 × 10^6^ cells per well) were cultured in 48-well vessels during 2–3 h and then washed for removal of non-adherent cells. Macrophages were infected or not with *T. cruzi* parasites (1 × 10^6^ metacyclic parasites per well) during 3-4 h before washing out extracellular parasites and additional non-adherent cells. Cultures were maintained in culture medium plus 10% FCS at 37 °C and 7% CO_2_ in a humid atmosphere. Trypomastigotes released from macrophages were counted in culture supernatants after 2–4 weeks of infection.

### T cells

Splenocytes were depleted of red blood cells by treatment with Tris-buffered ammonium chloride, followed by nylon wool filtration to obtain T-cell-enriched suspensions. Purified CD8 T cells were obtained by negative selection of T-cell-enriched suspensions with a mAb mix (containing anti-B220, anti-panNK, anti-MHC-II, anti-CD4, and anti-CD11b mAbs from BD Biosciences) and anti-rat IgG magnetic beads (Dynal, Oslo, Norway). Purified suspensions contained 85–95% CD8^+^ cells, as stained for detection of residual CD4 T cells and B cells with anti-CD19, anti-CD5, and anti-CD8 mAbs. In Figures 4a and b, we used 98% pure CD8 T cells positively selected by cell sorting with allophycocyanin- and PE-labeled anti-CD8 mAbs (53–6.7, eBioscience); by excluding CD4^+^ cells marked with FITC-labeled anti-CD4 mAb (GK1.5; BD Bioscience) in a MoFlo cytometer (Cytomation, Ft Collins, CO, USA).

### Macrophages cocultured with CD8 T cells

Purified CD8 T cells (1 × 10^6^ cells or 2 × 10^6^ cells per well) were added to infected macrophages (5 × 10^5^ or 1 × 10^6^ cells per well) in 48-well vessels and maintained in culture medium plus 10% FCS (0.5 ml per well). Some cocultures were set with CD8 T cells (3 × 10^6^/well) and non-infected macrophages (1.5 × 10^6^/well) in 24-well vessels. Cocultures were treated with 1 ng/ml IL-2 (BD Biosciences) to prevent spontaneous death^22^ and incubated with soluble anti-CD3 (5 *μ*g/ml). Cocultures were then treated or not with anti-FasL (10 *μ*g/ml) or control IgG mAb and maintained at 37 °C and 7% CO_2_ humid atmosphere during 48 h. Supernatants were collected for NO and cytokine assays. Lymphocytes were processed for apoptosis assays. Trypomastigotes released from infected macrophages were counted in culture supernatants after 2–4 weeks of infection.

### Treatment with anti-FasL

For *in vivo* inhibition of apoptosis, infected mice were injected intraperitoneally with 100 *μ*g of anti-FasL (MFL3 clone) mAb or control hamster IgG mAb (eBioscience) at 18 dpi. After 3 days, PECs were recovered with 4 ml of culture medium and centrifuged. Supernatants were kept for cytokine assays and PECs were processed for flow cytometry or cultured in 48-well vessels and infected or not with metacyclic trypomastigotes, as described above.

### Adoptive transfer of T cells

T-cell suspensions (5 × 10^6^/ml) from normal and infected (20 dpi) mice were stimulated *in vitro* with plate-bound anti-CD3 (10 *μ*g/ml) in the presence of zVAD (40 *μ*M) or 0.2% DMSO (control vehicle) during 4 or 24 h. After 4 h, T cells from infected mice were recovered from the plates, washed to remove zVAD or DMSO, and injected intraperitoneally (2 × 10^6^/0.4 ml) in infected mice (20 dpi). T cells that remained in cultures during 24 h were assayed for apoptotic CD4 and CD8 T cells. After 2 days, PECs were recovered from mice with 3 ml of DMEM. Upon centrifugation, supernatants were kept for cytokine assays and PECs were cultured in 48-well vessels and infected or not with metacyclic trypomastigotes, as described above. For flow cytometry, suspensions containing 1 × 10^6^ PECs were treated with 50 ng/ml of phorbol myristate acetate (PMA) (Sigma), 1 *μ*g/ml of ionomycin (Sigma), and 1 *μ*g/ml brefeldin (Golgiplug; BD Biosciences) in propylene tubes during 3 h before staining.

### Cytokine assays

Culture supernatants or diluted peritoneal exudates were used for cytokine and chemokine assays by sandwich ELISA, by using pairs of specific mAbs (R&D Systems or eBioscience), one of which was labeled with biotin, and developed with streptavidin-alkaline phosphatase (Invitrogen Life Technologies) and with *p*-nitrophenyl phosphate (Thermo Scientific Pierce, Waltham, MA, USA) as substrate. Cytokine levels were also analyzed in fresh supernatants by chemiluminescence arrays (Proteome Profiler – Mouse Cytokine Array Panel A Array Kit, R&D Systems), according to the manufacturer's instructions.

### Statistics

All tests were performed, by using the GraphPad Prism (v. 6.0). Results are expressed as average and S.E.M. in figures. The number (*N*) of animals per group is indicated in figure legends. For parasite load, data were transformed to log of parasites per ml for statistical analysis. Data were analyzed by Kolmogorov–Smirnov test for assessing normal distribution and by unpaired Student's two-tailed *t*-test or ANOVA, followed by Dunnett's or Tukey's post-test. Significant differences are indicated for *P*<0.05 (*), *P*<0.01 (**), *P*<0.001 (***), and *P*<0.0001 (****). For *in vitro* experiments, data are expressed as the average of technical replicates per treatment and S.E.M. When analyzing more than two groups or treatments, we performed ANOVA followed by Tukey's or Bonferroni post-test. Significant differences in *t*-tests and ANOVA are indicated as above.

## Abbreviations

Dpi: days post infection
FasL: Fas ligand
MGL1: macrophage galactose-type lectin-1
PEC: peritoneal exudate cell
PMA: phorbol myristate acetate
PGE-2: prostaglandin E-2
