# Supplementary material for: Apoptotic CD8 T-lymphocytes disable macrophage-mediated immunity to Trypanosoma cruzi infection
Source: Cell Death Dis. 2016 May 19;7(5):e2232–. doi: 10.1038/cddis.2016.135 (PMC4917666; doi:10.1038/cddis.2016.135)
Supplement: Supplementary Figure 1 [file cddis2016135x1.pdf]

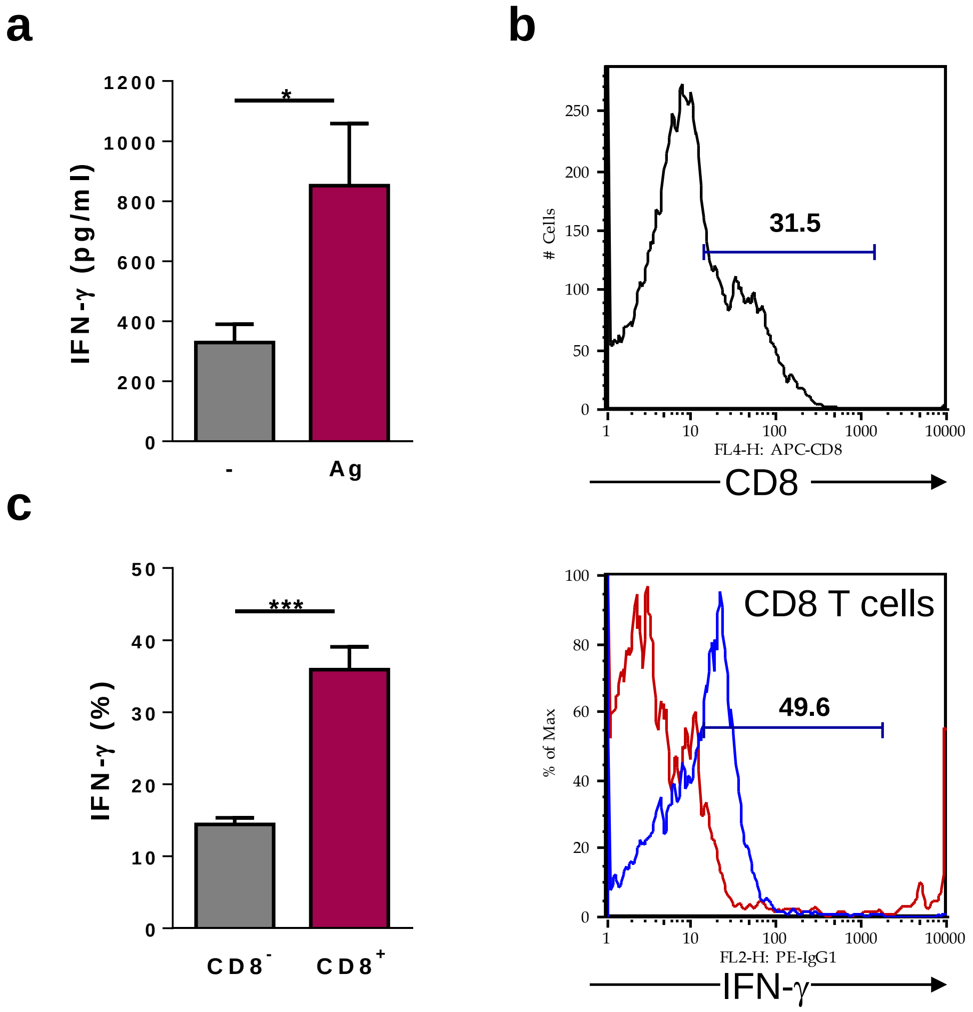

Figure S1. IFN-γ-producing CD8 T-cells from infected mice. PECs (2 x 10^6/well) from *T. cruzi*-infected mice (dpi) were adhered, genltly washed, and cultured with 10 μg/ml of a *trans*-sialidase Ag (IYNVGQVSI, Genescript, Piscataway, NJ, USA) able to bind H2-K^d (ref. 35). (**a**) IFN-γ was measured in 24-h supernatants by ELISA. (**b, c**) Cells were washed out, stained with APC-anti-CD8, followed by intracellular staining with PE-anti-IFN-γ or rat IgG1 mAb (BD Pharmingen). In **b**, stimulated CD8 T-cells were analyzed for IFN-γ expression (blue line) versus isotype control mAb (red line). Panel **c** depicts IFN-γ expression in both $CD8^-$ and $CD8^+$ cells. Significant differences are indicated (*) as analyzed by t test (n = 7 technical replicates) .
