# Supplementary material for: Apoptotic CD8 T-lymphocytes disable macrophage-mediated immunity to Trypanosoma cruzi infection
Source: Cell Death Dis. 2016 May 19;7(5):e2232–. doi: 10.1038/cddis.2016.135 (PMC4917666; doi:10.1038/cddis.2016.135)
Supplement: Supplementary Figure 2 [file cddis2016135x2.pdf]

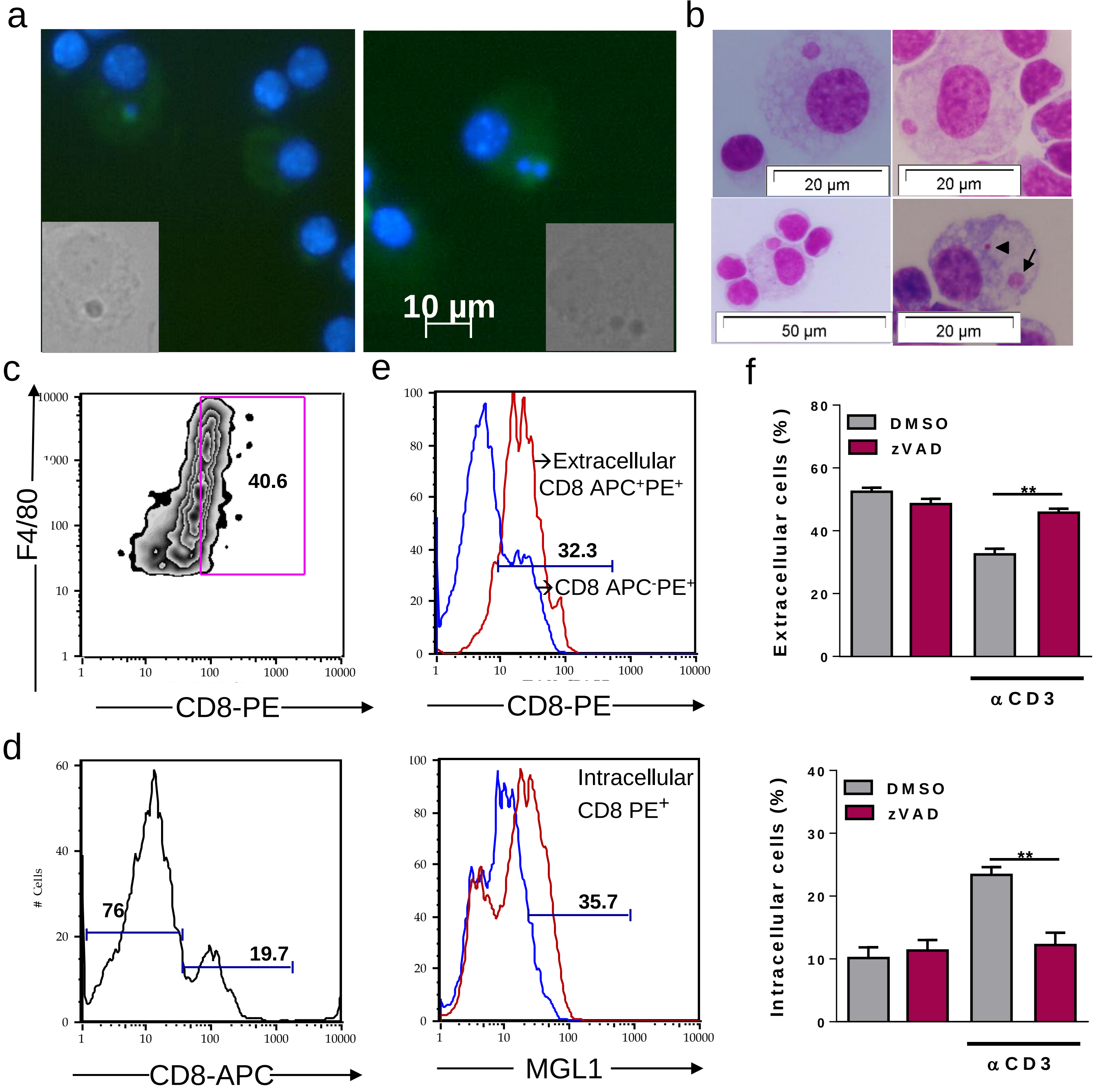
a
10 µm
b
20 µm
20 µm
50 µm
20 µm
c
F4/80
40.6
CD8-PE
d
Cells
76
19.7
CD8-APC
e
→Extracellular
CD8 APC⁺PE⁺
32.3
→CD8 APC⁻PE⁺
CD8-PE
Intracellular
CD8 PE⁺
35.7
MGL1
f
Extracellular cells (%)
DMSO
zVAD
**
α CD3
Intracellular cells (%)
DMSO
zVAD
**
α CD3

Figure S2. Efferocytosis by macrophages from infected mice. Efferocytosis by PECs from *T. cruzi*-infected mice in (**a-b**) cytospins (18-22 dpi) and (**c-f**) flow cytometry. (**a**) DAPI/MGL1 staining and bright field (**b**) panoptic staining. Inset: arrow indicates an apoptotic body and arrow head points to a *T. cruzi* parasite. (**c-f**) Macrophages bearing $CD8^+$ events. PECs were first stained for surface markers (APC-CD8, F4/80, MGL1/rat IgG2a), permeabilized, and then stained with PE-labeled anti-CD8 or rat IgG2a). (**c**) $F4/80^+$ macrophages were evaluated for intracellular PE-CD8 staining. Gate was based on isotype control mAb (PE-IgG2a, 7 %). (**d**) Extracellular (APC^+) CD8 cells and APC^{neg} cells were further analyzed in **e** for intracellular PE^+ CD8 staining. (**e**) Intracellular $CD8^+$ events in $MGL1^+$ macrophages. APC^+ CD8 T cells stains with PE-anti-CD8 and were used as a positive control (red line). Intracellular $CD8^+$ events within APC^{neg} cells (blue line) were gated and further analyzed for MGL-1 expression (red line) or control AF-IgG2a mAb (blue line) in bottom panel. (**f**) zVAD reduces lymphocyte apoptosis and efferocytosis by macrophages. Purified CD8 T cells (4 x 10^6/well) were treated with zVAD (80 µM or DMSO (0.4 %) and washed before coculture with PECS (1.5 x 10^6/well) from infected mice. Cocultures were stimulated or not with plate-bound anti-CD3 in triplicates. Upper panel depicts extracellular CD8 T cells and bottom panel shows intracellular $CD8^+$ events, evaluated as above. Significant differences are indicated (*) as analyzed by t test.
