# Supplementary material for: Apoptotic CD8 T-lymphocytes disable macrophage-mediated immunity to Trypanosoma cruzi infection
Source: Cell Death Dis. 2016 May 19;7(5):e2232–. doi: 10.1038/cddis.2016.135 (PMC4917666; doi:10.1038/cddis.2016.135)
Supplement: Supplementary Figure 3 [file cddis2016135x3.pdf]

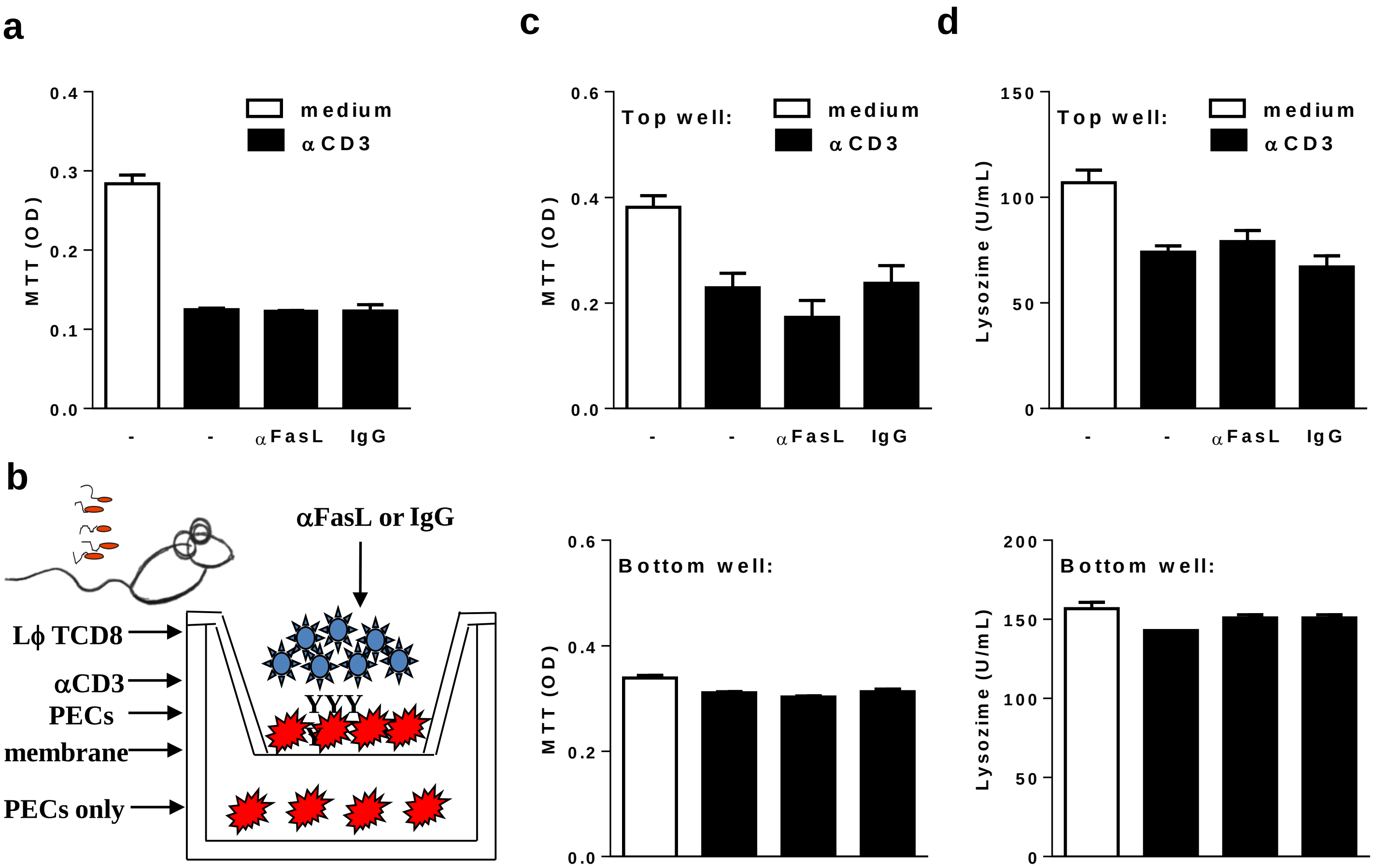

Figure S3. Activated CD8 T-cells induce FasL-independent macrophage killing. **(a-d)** Peritoneal macrophages from infected mice were infected **(a)** or not **(c, d)** with *T. cruzi* and cocultured (**a**; membrane inserts in **c,d**) or not (**c, d**, bottom wells) with purified splenic CD8 T cells from infected mice in the presence or absence of soluble anti-CD3. Cocultures in **a** or in top wells **(c, d)** were further treated or not with anti-FasL or control IgG in the presence of IL-2. After 48 h, supernatants were collected, CD8 T cells were washed out, and adherent cells were evaluated for viability by MTT assay (**a**, **c**). (**d**) Lysozyme release from macrophages was assessed in supernatants (Lysozyme Assay kit, Invitrogen). Data are expressed as the means and SEM of 2 or 3 technical replicates.
