# Supplementary material for: Apoptotic CD8 T-lymphocytes disable macrophage-mediated immunity to Trypanosoma cruzi infection
Source: Cell Death Dis. 2016 May 19;7(5):e2232–. doi: 10.1038/cddis.2016.135 (PMC4917666; doi:10.1038/cddis.2016.135)
Supplement: Supplementary Figure 4 [file cddis2016135x4.pdf]

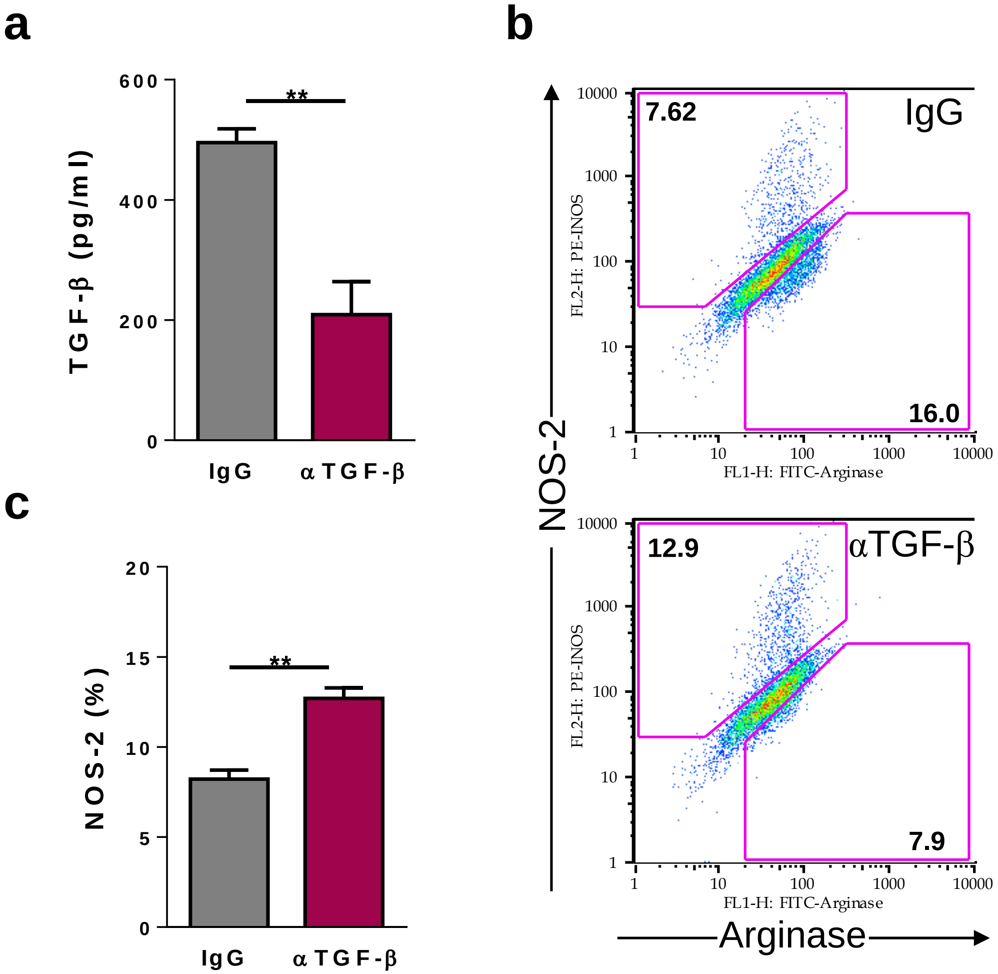

Figure S4. TGF-β suppresses NOS-2 expression by macrophages from infected mice. PECs from infected mice (20 dpi) were washed twice, plated, and cultured with medium supplemented with 1 % nutridoma (Roche, Mannheim, Germany) in triplicates. Cultures were treated with 10 µg/ml of anti-TGF-β (mAb 1835) or control mouse IgG1 mAb (R&D). After 48 h, (**a**) suppernatants were assayed for total TGF-β production by ELISA and (**b, c**) cells were detached and F4/80+ macrophages were evaluated for expression of NOS-2 and arginase 1, by intracellular staining with PE-anti-NOS-2 (ebioscience) and FITC-anti-arginase 1 (R&D). Gates were set by excluding unspecific staining with isotype control Abs (PE-rat IgG2a, FITC-sheep IgG). Panel **c** depicts NOS-2 expression. Significant differences are indicated (*) as analyzed by t test.
